# Changing malaria intervention coverage, transmission and hospitalization in Kenya

**DOI:** 10.1186/1475-2875-9-285

**Published:** 2010-10-15

**Authors:** Emelda A Okiro, Victor A Alegana, Abdisalan M Noor, Robert W Snow

**Affiliations:** 1 Malaria Public Health & Epidemiology Group, Centre for Geographic Medicine Research - Coast, Kenya Medical Research Institute/Wellcome Trust Research Programme, P.O. Box 43640, 00100 GPO, Nairobi, Kenya; 2 Centre for Tropical Medicine, Nuffield Department of Clinical Medicine, University of Oxford, CCVTM, Oxford OX3 7LJ, UK

## Abstract

**Background:**

Reports of declining incidence of malaria disease burden across several countries in Africa suggest that the epidemiology of malaria across the continent is in transition. Whether this transition is directly related to the scaling of intervention coverage remains a moot point.

**Methods:**

Paediatric admission data from eight Kenyan hospitals and their catchments have been assembled across two three-year time periods: September 2003 to August 2006 (pre-scaled intervention) and September 2006 to August 2009 (post-scaled intervention). Interrupted time series (ITS) models were developed adjusting for variations in rainfall and hospital use by surrounding communities to show changes in malaria hospitalization over the two periods. The temporal changes in factors that might explain changes in disease incidence were examined sequentially for each hospital setting, compared between hospital settings and ranked according to plausible explanatory factors.

**Results:**

In six out of eight sites there was a decline in Malaria admission rates with declines between 18% and 69%. At two sites malaria admissions rates increased by 55% and 35%. Results from the ITS models indicate that before scaled intervention in September 2006, there was a significant month-to-month decline in the mean malaria admission rates at four hospitals (trend P < 0.05). At the point of scaled intervention, the estimated mean admission rates for malaria was significantly less at four sites compared to the pre-scaled period baseline. Following scaled intervention there was a significant change in the month-to-month trend in the mean malaria admission rates in some but not all of the sites. Plausibility assessment of possible drivers of change pre- versus post-scaled intervention showed inconsistent patterns however, allowing for the increase in rainfall in the second period, there is a suggestion that starting transmission intensity and the scale of change in ITN coverage might explain some but not all of the variation in effect size. At most sites where declines between observation periods were documented admission rates were changing before free mass ITN distribution and prior to the implementation of ACT across Kenya.

**Conclusion:**

This study provides evidence of significant within and between location heterogeneity in temporal trends of malaria disease burden. Plausible drivers for changing disease incidence suggest a complex combination of mechanisms, not easily measured retrospectively.

## Background

Reports of declining incidence of malaria hospitalization, deaths and prevalence in several, diverse areas of Africa suggest that the epidemiology of malaria is in transition across the continent [1-17]. This transition has been coincidental with the scaling of intervention coverage and increased international funding for the control of malaria. However, precise attribution to intervention coverage alone remains circumstantial, not least because at most sites where declining disease incidence and prevalence have been reported, declines started before significant increase in donor assistance and scaled intervention coverage [2,8,9,12-14].

Adjusted trends in inpatient paediatric malaria case burden over 10 years in a sample of 17 hospitals with varied malaria transmission ecologies across Kenya were recently described using population-adjusted clinical data from defined hospital catchment areas [17]. The results showed divergent temporal patterns of disease incidence between sites. Importantly these data signalled that all was not equal across a single country. Here the possible mechanisms underlying the differences between these sites are explored in more detail in an effort to explain plausible drivers for changing disease incidence.

## Methods and Results

### Overview of sample selection, time-periods and methodological approach

For the purposes of evaluating mechanisms for change, hospital settings with adequate temporal data on insecticide-treated net (ITN) coverage (intervention change) and parasite prevalence (transmission intensity change) within defined spatial areas around hospitals that serve as the catchment to these hospitals were selected. Of the 17 original hospital settings the final selection of eight hospitals covered the dominant malaria ecologies that characterize Kenya including: three hospitals Bungoma, Kisumu, and Siaya District General Hospitals (DGH) in the Western/lakeside high transmission areas; Kericho and Kisii DGH located in the Highlands with typically epidemic transmission; and the three sites in Coastal Kenya: Malindi, Kilifi and Msambweni DGH.

Data have been assembled across two three-year time periods: September 2003 to August 2006 and September 2006 to August 2009: corresponding to important timelines for malaria intervention and drug policy change in Kenya. A range of plausible drivers and effect modifiers of changes in disease incidence were identified including intervention coverage, rainfall, service use and malaria transmission intensity. These data have been assembled at different spatial resolutions to match the communities served by the hospitals used to define disease incidence.

Defining attribution is fraught with many challenges and "plausibility designs" are often regarded as the only feasible option to evaluate the impact of nationally promoted large-scale intervention or changing risks [18,19]. These approaches have been recently promoted as a means of examining changes consequent upon scaled malaria control effort in sub-Saharan Africa [20]. Initially, temporal changes in factors that might explain changes in disease incidence were sequentially examined for each hospital setting, and were compared between hospital settings and plausible explanatory factors were ranked relative to proportional changes in disease incidence. An analytical procedure called *intervention analysis *was then used, this allows some exogenous event, in this case increasing ownership of ITNs and or a change in the first-line treatment policy to ACT, to occur that would affect the behaviour of the time-series of disease incidence being modelled as an ARMA process using the segmented regression [21,22].

### Defining the geographic scope of attribute data - hospital catchment populations

High resolution census data were used to produce population distribution estimates around each hospital and combined with a random sample of admissions where residence was defined to compute distance travelled from their homes to the hospital. Thematic maps were created in ARCGIS 9.1 (ESRI, Inc., Redland, CA, USA) and used to define a minimum catchment of census bureau enumeration areas (EA) that would capture more than 90% of all admissions to the paediatric wards of each hospital [17]. Population counts in each EA where then projected from the 1999 census using growth rate curves and corrected for the proportion of the population aged less than 15 years [23]. The 0-14 year projected population estimates were used to compute the person-years-at-risk (PYAR) for the mid years 2004/5 and 2007/8 to estimate PYAR in the 2003-2006 and 2006-2009 periods respectively.

### Paediatric admission data

Inpatient hospital registers were arranged serially to represent a continuous, uninterrupted series. Each admission entry in the registers was recorded on a tally sheet indicating the month of admission, whether a primary working diagnosis of malaria was assigned to the child and whether the child was aged less than 15 years. Admissions without a diagnosis of malaria were coded similarly. In six hospitals, slide-confirmed malaria diagnoses at admission were not universally available within and between hospital sites for the surveillance period, however most admissions are likely to have a blood film prepared pre-admission, but the parasitological results could not be linked to patient records or registers and this continues to pose a significant information gap. Therefore our working definitions of "malaria" were patients admitted with a diagnosis of malaria, probably managed clinically as malaria during their admission but without documented parasitological confirmation. At Siaya and Kilifi DGHs a more detailed clinical admission criteria have been used since 2003 and described elsewhere [24-26]. Data were missing for two months (September & October 2003) at Bungoma and admission numbers were modeled in STATA (version 10.1) by creating multiple imputed data sets for missing values using other existing variables (rainfall and the number of malaria and non-malaria deaths) for these missing data [17]. Malaria and non-malaria incidence was computed using the estimated children aged 0-14 years resident in the hospital catchment area and annualized over three years for the "baseline", pre-scaled intervention period September 2003-August 2006 and post-scaled intervention period September 2006-August 2009. Monthly malaria and non-malaria incidence rates were computed for each month of observation using the estimated annual proportion of the population aged less than 15 years.

**Figure 1:**
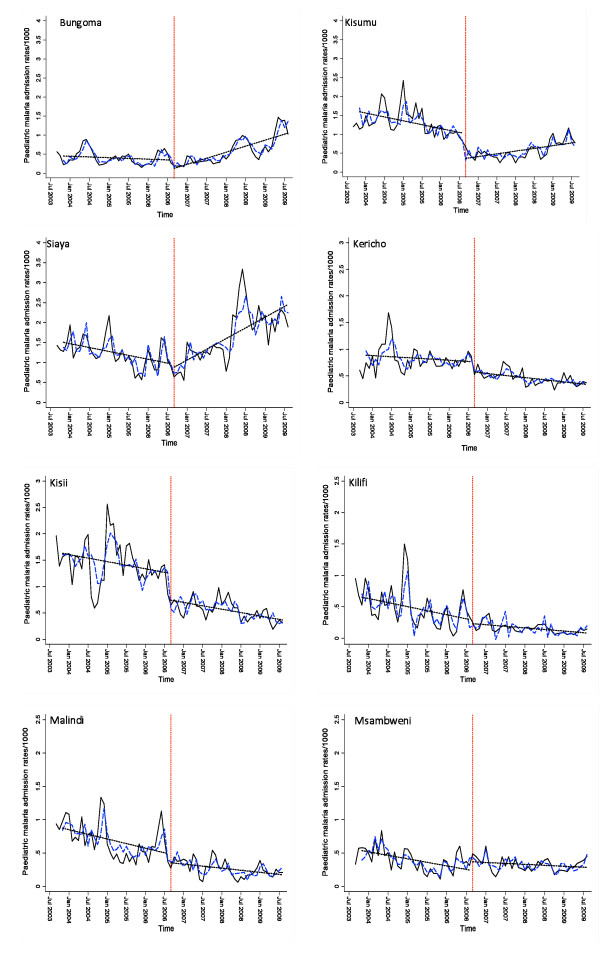
**Graph panels show changing paediatric hospitalizations rates due to malaria per 1 000 children 0-14 years before and after scaling up of interventions**. The data represents paediatric malaria admission rates by month (black solid line); model predictions of paediatric malaria hospitalization rates controlling for non-malaria case rates, rainfall and controlling for autoregressive and moving average effects (dashed blue line). Fitted lines illustrate the linear trends from model predictions (dashed line) for each segment of the period separated by a break point (red dashed vertical line).

An ARMAX model was then applied [21] with changes in service use (captured by non-malaria admission rates) included as an explanatory variable resulting in a predicted or smoothed malaria admission rate per month for each hospital site over the period 2003 to 2009 (Figure 1). The ARMAX model was then extended to include an interruption in the series to identify permanent changes in the level of the disease incidence series (interrupted time series analysis; ITS), that fits a linear regression to each segment, pre- and post- period of scaled intervention. The model is structured to identify changes in the level of the series [21,22,27], with intervention times entered as explanatory variables in the model. We have used an exact intervention time point corresponding to the intersection between the two time periods described above. Segmented regression analyses were then used to estimate the relative change in malaria admissions after scaled intervention compared with before, taking into account pre-ITN malaria admissions and trend fitting different means for each period.

During the six years of surveillance there were a total of 166,632 paediatric admissions including 78,530 (47%) admissions where a diagnosis included malaria. The proportion of admissions attributed to malaria across the entire surveillance period varied from 70% in Western/lakeside, 45% in the Highlands and 22% along the Kenyan Coast (Table 1).

**Table 1:** Temporally aggregated paediatric admission data for malaria each of the 8 hospitals between 2003- 2006 and 2006-2009 expressed per 1000 children aged 0-14 years at risk per annum and 95% confidence intervals computed using a Poisson distribution.

Hospital location	Average malaria admission rate/1000 Sep 2003 - Aug 2006 [95% CI] (number of malaria admissions)	Average malaria admission rate/1000 Sep 2006 - Aug 2009 [95% CI] (number of malaria admissions)	*Inc Rate Ratio (95% CI) *[Inc Rate Diff ] *(95% CI)*	Average non-malaria admission rate/1000 Sep 2003 - Aug 2006 [95% CI] (number of non-malaria admissions)	Average nonmalaria admission rate/1000 Sep 2006 - Aug 2009 [95% CI] (number of non-malaria admissions)	*Inc Rate Ratio (95% CI) *[Inc Rate Diff ] *(95% CI)*
**Western/Lakeside**						

Bungoma DGH	4.66 [4.53-4.79] (4,853)	7.21 [7.06-7.37] (8,500)	1.55 (1.50-1.61) [2.56] (2.36-2.76)	3.63 [3.52-3.75] (3,783)	3.45 [3.34-3.56] (4,063)	0.95 (0.91-0.99) [-0.19] (-0.34- -0.03)

Kisumu DGH	15.89 [15.61-16.18] (12,128)	6.81 [6.64-6.99] (5,578)	0.43 (0.42-0.44) [-9.08] (-9.41- -8.74)	3.28 [3.15-3.41] (2,501)	3.28 [3.16-3.41] (2,685)	1.00 (0.95-1.06) [0.00] (-0.18- 0.18)

Siaya DGH	14.93 [14.52-15.35] (5,042)	20.12 [19.65-20.60] (6,982)	1.35 (1.30-1.40) [5.19] (4.56-5.82)	8.10 [7.80-8.41] (2,736)	7.22 [6.94-7.51] (2,506)	0.89 (0.84-0.94) [-0.88] (-1.30- -0.47)

**Highlands**						

Kericho DGH	9.80 [9.55-10.05] 5,923	5.50 [5.32-5.68] (3,580)	0.56 (0.54-0.59) [-4.30] (-4.30- -3.99)	7.21 7.00-7.43 4,360	6.58 [6.38-6.78] (4,281)	0.91 (0.87-0.95) [-0.64] (-0.93- -0.35)

Kisii DGH	17.45 [17.12-17.79] (10,450)	6.60 [6.40-6.80] (4,201)	0.38 (0.36- 0.39) [-10.85] (-11.24- -10.46)	16.67 [16.35-17.00] (9,983)	17.28 [16.95-17.60] (10,995)	1.04 (1.01-1.06) [0.60] (0.15-1.06)

**Coastal**						

Kilifi DGH	5.98 [5.78-6.18] (3,345)	1.85 [1.75-1.97] (1,139)	0.31 (0.29-0.33) [-4.12] (-4.35- -3.89)	19.61 [19.24-19.98] 10,974	19.10 [18.75-19.45] (11,730)	0.97 (0.95-1.00) [-0.51] (-1.01- -0.00)

Malindi DGH	8.28 [7.96-8.61] (2,573)	3.21 [3.03-3.41] (1,105)	0.39 (0.36-0.42) [-5.07] (-5.44- -4.70)	18.76 [18.28-19.25] 5,828	16.88 [16.45-17.32] (5,807)	0.90 (0.87-0.93) [-1.88] (-2.53- -1.23)

Msambweni DGH	4.74 [4.51-4.97] (1,655)	3.91 [3.71-4.12] (1,476)	0.82 (0.77-0.89) [-0.83] (-1.13- -0.53)	7.15 [6.88-7.44] 2,498	8.93 [8.63-9.24] (3,372)	1.25 (1.19-1.32) [1.78] (1.37-2.19)

The annualized rate of paediatric malaria admission declined in the second observation period compared to the first at three locations on the coast (Table 1); the absolute decline (expressed as the incidence rate difference of malaria per 1000 between the periods 2003-2006 and 2006-2009) was greatest in Malindi (-5.07) and lowest in Msambweni (-0.82). These are equivalent to decline of 69% in Kilifi, 61% in Malindi to the smallest drop at 18% in Msambweni (Table 1; incidence rate ratio (IRR)). Reductions in the second period compared to the first were also observed for the two sites in the highlands Kericho (-4.30) and Kisii (-10.85) and one urban site close to Lake Victoria, Kisumu (-9.08) (Table 1). Conversely, malaria admissions rates increased by 55% and 35% at Bungoma and Siaya respectively (IRR; Table 1). The incidence rate differences per 1000 children 0-14 between the periods 2003-2006 and 2006-2009 in non-malaria admission rates were minimal at all sites except Malindi and Msambweni where the differences were 1.9 and 1.78, respectively (incidence rate difference; Table 1).

The relationship between malaria admissions by month shows declines in Kilifi, Malindi, Msambweni, Kisii, Kericho and only in the first period in Kisumu (Figure 1). Importantly the time trends show that the declines started before scaled intervention in Kilifi, Malindi, Kisii, Kericho and Kisumu. This appears less clear in Msambweni. Conversely the trend in malaria admission rates showed a rise at Bungoma and Siaya with a rise in cases occurring largely in 2008 and 2009. At Kisumu, somewhat of an outlier, following the decline in the first period there was a rise in malaria admission rates in the second period occurring in 2009 (Figure 1). Results from the interrupted time series analysis indicate that just before the beginning of the observation period, malaria admission rates on average were between 2.21 to 12.5 per 1000 children 0-14 per month (mean 6.30). Before September 2006, there was a significant month-to-month change in the mean number of malaria admission rates in Kisumu, Kilifi, Malindi and Msambweni with a significant downward trend (P-value for baseline trend <0.05) (Table 2). Immediately after the scaled-intervention, the estimated mean admission rates for malaria was significantly less by 0.52, 0.59, and 0.13 in Kisumu, Kisii and Msambweni but were not significant at the other sites. There was no significant change in the month-to-month trend in the mean admission rates for malaria across most of the hospitals except in Bungoma, Siaya , Kisumu and Msambweni where the month-to-month trend in the mean malaria admission rates increased significantly in the second period (P-value for trend change <0.05) (Table 2).

**Table 2:** Parameter estimates, confidence intervals and P-values from the full segmented ARMAX regression models predicting mean monthly numbers of malaria cases per month in eight hospitals over time

		Mean before scale up	Baseline trend	Mean after scale up	Trend change after scale up	
**Western/Lakeside**						

	Coefficient	2.2189	-0.0038	-0.0797	0.0251	
Bungoma DGH	95% CI	-5.8433 -	-0.0185 -	-0.4578 -	0.0043 -	
	*P-value*	0.2811	0.0109	0.2985	0.0459	
		*0.5900*	*0.6100*	*0.680*	***0.018***	

		12.5782	-0.0213	-0.5247	0.0292	
Kisumu DGH	Coefficient	5.7537 -	-0.0341 - -	-0.8117 - -	0.0062 -	
	95% CI	19.4028	0.0086	0.2377	0.0521	
	*P-value*	***0.000***	***0.001***	***0.000***	***0.013***	

		7.3604	-0.0125	-0.1275	0.0557	
Siaya DGH	Coefficient	-7.9641 -	-0.0405 -	-0.9069 -	0.0138 -	
	95% CI	22.6849	0.0156	0.6519	0.0975	
	*P-value*	*0.347*	*0.384*	*0.749*	***0.009***	

**Highlands**						

		2.9036	-0.0043	-0.1847	-0.0014	
Kericho DGH	Coefficient	-3.1163 -	-0.0156 -	-0.5648 -	-0.0210 -	
	95% CI	8.9234	0.0070	0.1954	0.0182	
	*P-value*	*0.344*	*0.457*	*0.341*	*0.888*	

		6.10113	-0.00892	-0.59444	0.00019	
Kisii DGH	Coefficient	-0.3973 -	-0.0209 -	-1.0789 - -	-0.0148 -	
	95% CI	12.5996	0.0031	0.1100	0.0152	
	*P-value*	*0.066*	*0.146*	***0.016***	*0.980*	

**Coastal**						

		7.2789	-0.0131	-0.0382	0.0094	
Kilifi DGH	Coefficient	3.3066 -	-0.0207 - -	-0.3036 -	-0.0069 -	
	95% CI	11.2512	0.0056	0.2272	0.0256	
	*P-value*	**0.000**	***0.001***	*0.778*	*0.260*	

		7.6455	-0.0134	-0.0587	0.0072	
Malindi DGH	Coefficient	2.6559 -	-0.0229 - -	-0.3656 -	-0.0111 -	
	95% CI	12.6351	0.0040	0.2483	0.0255	
	*P-value*	***0.003***	***0.005***	*0.708*	*0.441*	

	Coefficient	4.3503	-0.0078	-0.1287	0.0143	
Msambweni DGH	95% CI	2.7546 -	-0.0108 - -	-0.2452 - -	0.0080 -	
	*P-value*	5.9459	0.0048	0.0122	0.0206	
		***0.000***	***0.000***	***0.030***	***0.000***	

### Rainfall

To examine the effects of rainfall, an important determinant of malaria transmission, monthly precipitation data were obtained from meteorological stations located within the catchment areas of six hospitals. Because records were incomplete or unavailable, rainfall data was obtained from the nearest possible metrological station with complete data for Msambweni, Bungoma, Kilifi and Siaya (32 km to 70 km from the catchment boundary respectively). Continuous mean monthly and annual rainfall and the number of months in a year with rainfall >80 mm or >60 mm among other variables were examined. There was an increasing trend toward higher annual precipitation in the second observation period (2006-2009) compared to the first observation period (2003-2006). Between September 2006 and August 2009, the mean monthly rainfall in seven out of the eight study sites was higher on average than the three years prior to this period (Table 3 and 4). The mean difference in average monthly rainfall between the first and second observation periods was 19.5 mm (median 19.2; IQR 11.2-25.9). In addition, the number of three-month periods of continuous rainfall above 60 mm indicates that the period before 2006 was notably drier than the period after 2006 (Additional file 1 Table S1).

**Table 3:** Characteristics of Hospital Catchment areas 2003-2006

HOSPITAL	Projected Population <15 years in 2003	Average annual number of Total admissions (2003 - 2006)	Average annual Rainfall (mm) at Baseline (2003-2006)	**Median Age-Corrected Parasite Prevalence Jan 2002-Aug 2006 **^**1**^	ITN Coverage	**SP Success Rate Average % ACPR d14 (2000-2003)**^**2**^	**Number reporting fever in last 14 days DHS 2003**^**3**^	**Number (%) of fevers seeking any treatment from a Public Health Facility DHS 2003**^**3**^
**Western/Lakeside**								

Bungoma DGH	326137	2879	1337.0	15.3%	20.5%			
			
Kisumu DGH	245600	4876	1241.2	70.6%	26.2%	55.4-83.1	733^4^	168(22.9) ^4^
			
Siaya DGH	111026	2593	1241.2	54.7%	21.0%			

**Highlands**								

Kericho DGH	194090	3428	2130.5	5.2%	14.3%	70.37	718^5^	190(26.5)^5^
			
Kisii DGH	193608	6811	1978.0	22.2%	35.6%			

**Coastal**								

Kilifi DGH	178045	4773	1136.0	12.9%	20.5%			
			
Malindi DGH	98401	2800	954.7	11.8%	8.8%	67.2- 96.8	262	100(38.2)
			
Msambweni DGH	111917	1384	588.7	10.4%	15.1%			

Notes:

SP - Sulphadoxine-pyrimethamine ACPR - Acceptable Clinical & Parasitological Responses by day 14

^1^Includes data from surveys done in 2002 and school surveys done in January and February 2010 in Bungoma, Kisumu, Siaya and Kericho areas

^2 ^Study references: [46]; [36,47].

^3 ^Estimates reported at the Provincial level.

^4^Combined estimates from Western and Nyanza provinces

^5^Combined estimates from Rift Valley and Nyanza provinces

**Table 4:** Characteristics of Hospital Catchment areas 2006-2009

HOSPITAL	Projected Population <15 years in 2003	Average annual number of Total admissions (2003 - 2006)	Average annual Rainfall (mm) at Baseline (2003-2006)	Median Age-Corrected Parasite Prevalence Jan 2002-Aug 2006 ^1^	ITN Coverage	**SP Success Rate Average % ACPR d14 (2000-2003)**^**2**^	**Number reporting fever in last 14 days DHS 2003**^**3**^	**Number (%) of fevers seeking any treatment from a Public Health Facility DHS 2003**^**3**^
**Western/Lakeside**								

Bungoma DGH	417832	4188	1673.3	44.9%	36.0%			
			
Kisumu DGH	282645	2754	1472.1	29.8%	47.3%	93.4- 100.0	686^4^	210 (30.6)^4^
			
Siaya DGH	111026	2593	1241.2	54.7%	21.0%			

**Highlands**								

Kericho DGH	225159	2620	2095.4	0.01%	32.0%	N/A	205^5^	205 (35.7)^5^
			
Kisii DGH	218698	5065	2063.0	1.9%	51.1%			

**Coastal**								

Kilifi DGH	214441	4290	1388.6	3.7%	38.1%			
			
Malindi DGH	120658	2304	1145.6	4.5%	49.9%	87.0- 100.0	173	64 (37.0)
			
Msambweni DGH	111917	1384	588.7	10.4%	15.1%			

Notes:

AL - Artmether-lumefathrine

ACPR - Acceptable Clinical & Parasitological Responses. Day 28 PCR corrected parasitological and clinical failure rates

^1^Includes data from school surveys done in January and February 2010 in Bungoma, Kisumu, Siaya and Kericho areas

^2^Study references: [48-53]

^3^Estimates reported at the Provincial level. These data were available at the provincial level and not specific to each district or catchment area and are presented as such.

^4^Combined estimates from Western and Nyanza provinces

^5^Combined estimates from Rift Valley and Nyanza provinces

### Parasite prevalence

One of the most widely used measures of *Plasmodium falciparum *malaria transmission intensity is the parasite rate (*Pf*PR) measured through community-based surveys and expressed as the proportion of people infected at one point in time. A comprehensive dataset has been assembled of all geo-located *Pf*PR survey data undertaken in Kenya since 1974 [28]. These data have been re-sampled to identify surveys undertaken within each hospital catchment, between 2002 and 2010 where more than 50 people had been surveyed. The age-ranges reported varied between surveys and these were standardized to a single age range 2-10 years (*Pf*PR_2-10_) using algorithms described elsewhere [29]. The prevalence estimates across the two time periods were summarized using the median and inter-quartile ranges of age standardized prevalence estimates (*Pf*PR_2-10_) across the 300 surveys identified as having been undertaken within the catchment areas since 2002 (Additional file 1 Table S2).

During the first observation period, before scaled intervention coverage in September 2006, the median *Pf*PR_2-10 _was highest around Lake Victoria. At Kisumu median *Pf*PR_2-10 _was 71% and around Siaya DGH *Pf*PR_2-10 _was 55% (Table 3), however it was reportedly lower in Bungoma during this period (median *Pf*PR_2-10_, 15%; Table 3). In the highlands median *Pf*PR_2-10 _was 22% at Kisii and 5% at Kericho (Table 3). Along the Kenyan coast median *Pf*PR2-10 was between 10 and 13% during the first, pre-scaled intervention period (Table 3). By the second period (2006-2009) median *Pf*PR_2-10 _had declined in six out of the eight sites, this decline varied between sites (Table 4) and the median *Pf*PR_2-10 _had increased to over 45% at Bungoma and rose slightly at Msambweni from 10% to 12% (Table 4). During the second observation period two hospital catchment areas had a median *Pf*PR_2-10 _of greater than 40%: Bungoma and Siaya (Table 4).

### ITN coverage

In 2001, the Kenyan Government adopted a policy on ITN to ensure 60% coverage by 2010 [30]. The dominant ITN delivery approach between 2003 and September 2006 was a combined full cost recovery retail sector promotion and subsidized cost ITN distribution using social marketing at rural clinics [31]. In 2006 the Ministry of Health, using funds from the Global Fund, launched a large-scale distribution campaign of free ITN to children under the age of five years, which was completed in September 2006 and rapidly increased equitable coverage of ITN nationwide [31]. Various household survey data have been collected in Kenya since 2003 aimed at defining the proportion of children below the age of five sleeping under an ITN during the night before the survey. Cluster level data congruent with the catchment areas defined for each of the eight hospitals were re-sampled from the national Demographic and Health Survey (DHS) undertaken in 2003 [32], Population Services International household sample surveys undertaken in 2005 and 2007 [33] and a national malaria indicator survey [34] undertaken in 2007. Each cluster was allocated to a time-period and data summed across clusters within each catchment. During the 2003 DHS survey only data on children was available but subsequent surveys included information on all age-groups and have therefore focussed on all-age use of ITN for comparisons between first and second observation periods.

During the first observation period bed net use by children aged less than five was universally low, the highest average coverage in 2003 was recorded in Kisumu (0.29%). The average level of coverage across all eight sites was 20.3% in 2005 with the lowest level of coverage recorded in Malindi at 9%, in Kericho and Msambweni coverage was 14% and 15% respectively while the highest estimated level of coverage was in Kisii at 37%. By 2007, one year after the free mass distribution campaigns ITN coverage had increased considerably to an estimated average coverage of 41% across all sites ranging from lowest coverage in Kericho at 32% to high of 50% in Malindi and 51% in Kisii. The average percentage increase was 21%, however at Bungoma, Siaya, Kilifi and Kericho the increase in coverage was less than 20% and remained below 40% in all 4 sites including Msambweni during the post-scaled coverage period (Additional file 1 Table S3).

### Drug policy change and access to efficacious antimalarials

Between 2000 and April 2006, sulphadoxine-pyrimethamine (SP) was the only drug available in most government clinics [35] however, the efficacy of SP declined rapidly over this period including empirical evidence at some of the selected hospital sites that demonstrated success rates of less than 75% by day 14 following treatment (Table 3, [36]; [37]). Officially first line treatment policy was changed in April 2004 to the more efficacious artemether-lumefathrine (AL) but the new policy was not effectively implemented until September 2006 [38]. The clinical and parasitological efficacy of AL was in excess of 94% in 2002-2003 at the coast [39] and several clinical trials undertaken since 2006 have shown AL efficacy to be in excess of 87%. Information on SP and AL sensitivity was attributed to regions that broadly cover the catchments included in the analysis but it was not possible to re-assemble these data specifically to the communities served by the hospitals (Table 3 and 4).

Prior to 2006 SP was ubiquitously available in government facilities and through private retail providers [35,38]. However, AL implementation in 2006 limited access to only government and mission facilities [40] and the supply to these facilities has been erratic and incomplete [41]. Access to anti-malarials within 48 hours of onset of symptoms was generally poor across Kenya throughout the period of observation 2003-2009. At four sentinel districts (including Msambweni and Kisii), Amin and colleagues showed that use of any anti-malarial within 48 hours by febrile children was 15% in 2001 and 17% in 2006 [42]; unpublished data) and in the same sites this rose to only 23% by 2007 [43].

Household survey data on drug use is much less prolific than ITN usage data and, therefore, it was only possible to assemble national survey data at wider spatial resolutions than the catchment areas in the eight hospital sites. The respective provincial level data on treatments with an anti-malarial for children who had a reported fever in the last 14 days were assembled from the DHS 2003 survey and the MIS in 2007 to represent drug access in the two observation periods (Table 3 and 4). Between 2003 and 2006 the proportion of children with fever in the last two weeks who sought treatment from a Public Health Facility was estimated to be 23% in Western/Lakeside region, 27% in the Highlands and was highest at 38% on the Coast (Table 3). By the second period, four years after the survey, access to treatment had increased slightly across two study regions with the proportion of fevers seeking treatment from a Public Health Facility reported to be 31% in the Western/Lakeside and 36% in the Highlands. However, on Coast access to treatment has remained largely unchanged at 37% in 2007 (Table 4).

### Comparison of health indictors pre- and post 2006

The surveillance data were analysed by time to determine whether patterns in hospitalized malaria case incidence could be associated to equivalent changes in several important covariates. Outcome indicators are summarized as incidence rate ratios and incidence rate differences based on admission events with mid-period under 15 population estimates as the denominator. The relative change and the absolute changes (95% CI) in covariates between period 1 and 2 calculated for each hospital were used as the comparative measures. The absolute change is used to describe the actual increase or decrease from the estimate in period one and is used to explore the magnitude of change. Relative change is used to describe changes between period one and two by comparing the absolute change to the reference value which here is the value of the estimate in the first period and has been used here to explore processes of causation specifically to examine the effect of changes in intervention coverage or climate variables on the occurrence of malaria cases. We examine changes in relation to the starting endemicity and baseline ITN coverage for the parasite prevalence and ITN coverage respectively.

**Figure 2:**
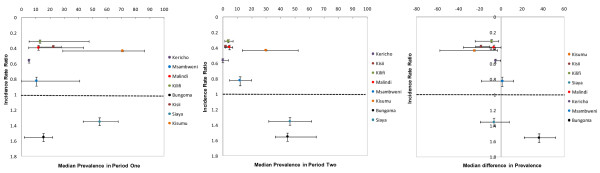
**Relationship between changes in the incidence rate of malaria admissions between period one and two (Incidence rate ratio) and equivalent absoulte changes in transmission intensity across 8 hospital sites in Kenya (Left-starting *Pf*PR; Middle - *Pf*PR at the end of the study and Right - absolute difference in prevalence between period one and two)**.

**Figure 3:**
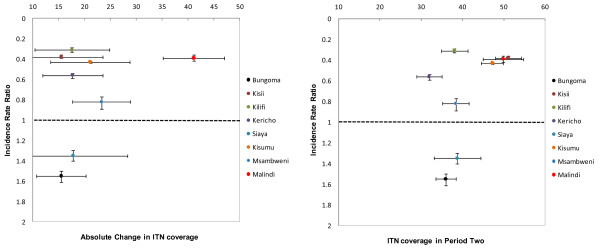
**Relationship between changes in the incidence rate of malaria admissions between period one and two (Incidence rate ratio) and equivalent absolute changes in ITN coverage across 8 hospital catchments (Left- absolute difference in ITN coverage between period one and two; Right - ITN coverage in the follow-up period)**.

Plausibility patterns exploring factors that might explain changes in disease incidence across hospital settings represented by different colors are shown in Figures 2 and 3. Patterns in IRR (x-axis) were examined against parasite prevalence in the first and second period, the absolute change in prevalence, ITN coverage in the second period and the absolute change in ITN coverage (x-axis). No obvious patterns were clearly evident however, considering that rainfall increased during the same period, out of all the factors we have examined, there is a suggestion that sustained high transmission intensity in the second period (Figure 2; middle graph) and the magnitude of change in ITN coverage (Figure 3; right graph) might explain some of the variation in effect size.

In addition, the combined effect of various factors were examined using the ranking of plausible explanatory factors (ranked by magnitude) with 1 representing the highest score/change and 8 the lowest score/change relative to the ranking of the effect size represented as the absolute change in disease incidence in each hospital site (Table 5). Despite the fact that the ranking shows largely ambiguous patterns; there is some indication that transmission intensity reduced to low levels in the sites that witnessed a substantial drop in malaria hospitalization (ranking 1-5) with the exception of Kisumu and Msambweni (Table 3 and 4)_. _The ranking tables also showed that the two sites with a rise in malaria hospitalization appear to have the highest transmission intensity in the second period (ranked 1 and 2); (Table 5). Kisii which had the highest absolute drop in malaria admission rates also had the highest level of ITN coverage in period two and a significant drop in *Pf*PR_2-10 _(ranked 2).

**Table 5:** Plausibility ranking table indicating the ranking of each hospital by absolute change in several important factors

		Rank by Covariates							
**Absolute Change in Incidence**		**Parasite Prevalence**			**ITN Coverage**		**Rainfall**		

	**Rank by IRD**	***Pf*PR2-10 2003 -2006**	***Pf*PR2-10 2006-2009**	**Change in *Pf*PR2-10**	**ITN coverage 2006- 2009**	**Change in ITN coverage**	**Change in 3 Continuous months with RF >60 mm**	**Change in Total RF**	**Change in mean monthly RF**
Kisii (-10.85)	1	3	7	2	1	7	**8**	7	7
Kisumu (-9.08)	2	1	3	1	3	3	2	4	4
Malindi (-5.07)	3	6	5	4	2	1	1	6	6
Kericho (-4.30)	4	8	8	6	8	5	6	**8**	**8**
Kilifi (-4.12)	5	5	6	3	6	6	5	3	2
Msambweni (-0.83)	6	7	4	**7**	5	2	6	2	3
Bungoma (2.56)	7	4	2	**8**	7	7	4	1	1
Siaya (5.19)	8	2	1	5	4	4	2	4	4

Notes:

**IRD**-Incidence Rate Difference

1 = highest value/change 8 = smallest value/change

Bold numbers represents instances where the observed trend is the reverse of the common trend i.e. an increase in transmission intensity (*Pf*PR_2-10 _) or a reduction in rainfall

## Discussion

The temporal patterns of paeditaric malaria admissions at eight hospitals across the diverse malaria transmission ecology of Kenya were examined between two 36 month periods September 2003 and August 2009 and the period September 2006 to August 2009 representing two periods best described as reflecting major shifts in intervention policy change and scaled intervention. At six hospital sites (Kisumu, Kericho, Kisii, Kilifi, Malindi and Msambweni) hospitalization from malaria showed a significant reduction between these two time periods (Table 1), but in four cases the decline started before scaled intervention (significant baseline downward trend in Kilifi, Malindi Msambweni, and Kisumu, Table 2). Similar declines were observed at Msambweni on the southernmost tip of the Kenyan coast but these declines were less marked. Kisumu represents an outlier where despite the fact that there was a significant drop in malaria cases between period one and two hospitalization from malaria showed a significant upward trend in period two. At Siaya and Bungoma paediatric malaria admission rates rose in the second period compared to the pre-scaled intervention period before September 2006. It is important to acknowledge that the majority of the data analyzed here are not based upon parasitological confirmed cases of malaria as these data were often not available and of variable quality and accuracy and may have contributed to some systematic bias in the data and remains an inherent methodological limitation.

A number of extrinsic (rainfall), biological (transmission intensity) and intervention (ITN coverage and ACT access) that may have explained the changes in the interupted time-series of paediatric malaria admissions were examined. Rainfall proved not to explain any of the changes directly as the second observation period was wetter than the first at all sites where declines occurred and changes in rainfall patterns were not different between sites with increasing malaria admissions compared to those without (Table 3 and 4). Parasite prevalence during the period prior to scaled intervention among communities located within the catchments of the hospitals signalled that there was some tendency for those areas that showed the largest declines in admission rates in post-intervention to be of lowest transmission intensity in the period prior to scaled intervention for some but not all of the sites. For example Bungoma and Kisumu were ranked low and high *Pf*PR_2-10 _in the first observation period. Theoretically it has been suggested that scaled intervention coverage with ITN is least likely to impact on disease incidence where starting transmission intensity is highest [44]. These data do not rule out this possibility with largest differences observed in admission rates among areas where starting *Pf*PR_2-10 _was low but this trend was not consistent across the eight sites (Table 3; Figure 2). It was not possible to re-assemble spatially congruent data on drug sensitivity and drug access as a driver of temporal changes in disease incidence; nevertheless the data do show a marginal increase in the use of public health services between the two observation periods but must be balanced with the periennial problems of poor drug availability in this sector. It seems unlikely that changing to an ACT policy in September 2006 would have been a major factor in the changing incidence of malaria admissions. However, it was possible to re-construct site-specific data on ITN coverage and the changes in population coverage with an ITN were dramatic between the two observation periods (Tables 3 and 4). The absolute decline in malaria admission rates was highest in the three areas where post-scaled ITN coverage was highest (Table 3: Kisii, Kisumu and Malindi) and two of these sites also showed the highest declines in *Pf*PR_2-10 _(Table 3: Kisii and Kisumu). Two areas where ITN coverage remained poor post-September 2006 were Bungoma and Siaya where malaria admissions increased. These observations at these sites are largely consistent with expectations [45], but there were also anomalies to this observed pattern. These anomalies do not imply that increased ITN coverage has not impacted on disease incidence rather that the explanations for temporal changes in admissions are complex and unlikely to be a result of ITN alone.

## Conclusions

Support for the complex nature of change derives from the observation that most sites where declines between observation periods were observed showed evidence of changing admission rates ahead of the free mass ITN distribution campaign and prior to the implementation of ACT across Kenya. What then are the drivers of this epidemiological transition? In this paper an attempt was made to retrospectively assemble as much information as possible to develop a plausibility framework around an interrupted time-series data analysis. The conclusion is that although there have been changes, this has not been consistent across sites, both starting endemicity and ITN coverage may have accelerated declines but it is not possible to confidently identify unique factors associated with the trend in malaria admissions. In part this may well stem from having to depend on retrospective, opportunistic data assemblies to explain change. This is an unfortunate position to be in and a lesson for future monitoring and evaluation of epidemiological transitions. Without more prospectively designed plausibility studies with carefully assembled disease data congruent with information on plausible explanatory variables for change it may never be understood what is driving the malaria transition in Africa.

## Competing interests

The authors declare that they have no competing interests.

## Authors' contributions

EAO was responsible for study design, data cleaning, analysis, interpretation and production of the final manuscript. VAA assisted in the primary assembly of the covariate data at each of the 8 hospitals. AMN provided temporal estimations of *Pf*PR_2-10 _in each of the catchment areas and contributed to the final manuscript. RWS was responsible for overall scientific management, analysis, interpretation and preparation of the final manuscript. All authors read and approved the final manuscript.

## Supplementary Material

Additional file 1 **Characteristics of hospital-catchment sites - Changes in Plausibility Drivers**. The data provided represent the analysis of temporal changes in factors that might explain changes in disease incidence in each hospital setting and compared between hospital settings. Click here for file
