# Supplementary material for: Changing malaria intervention coverage, transmission and hospitalization in Kenya
Source: Malar J. 2010 Oct 15;9:285. doi: 10.1186/1475-2875-9-285 (PMC2972305; doi:10.1186/1475-2875-9-285)
Supplement: Additional file 1 — Characteristics of hospital-catchment sites - Changes in Plausibility Drivers. The data provided represent the analysis of temporal changes in factors that might explain changes in disease incidence in each hospital setting and compared between hospital settings. [file 1475-2875-9-285-S1.DOC]

**Additional Information**

**AI Table S1:** Rainfall Patterns and Summaries across the eight study sites between 2003-2006 and 2006-2009

| **Hospital** | **Total precipitation (mm)**  **Average monthly rainfall (mm)**  **[Number of months with rainfall >60mm, > 80mm]**  **(Number of months 3 continuous months with rainfall > 60mm)**  **Sept 2003-Aug 2006** | **Total precipitation (mm)**  **Average monthly rainfall (mm)**  **[Number of months with rainfall >60mm, > 80mm]**  **(Number of months 3 continuous months with rainfall > 60mm)**  **Sept 2006-Aug 2009** | ***Relative Change (%)^2^***  ***Total precipitation***  **Absolute Change Avg monthly rainfall**  **[Absolute difference in number of months with rainfall >60mm ]^3^** |
| --- | --- | --- | --- |
| **Western/Lakeside** |  |  |  |
| Bungoma DGH | 4011.0  111.42  [25,22]  (11) | 5020.0^4^  139.44  [30,25]  (22) | 25.2  28.02  [11] |
| Kisumu DGH^1^ | 3723.7  103.44  [22, 22]  (7) | 4416.3  122.68  [28, 24]  (19) | 18.6  19.24  [12] |
| Siaya DGH^1^ | 3723.7  103.44  [22, 22]  (7) | 4416.3  122.68  [28, 24]  (19) | 18.6  19.24  [12] |
| **Highlands** |  |  |  |
| Kericho DGH | 6391.5  177.54  [33, 29]  (25) | 6286.2  174.62  [31, 28]  (26) | -1.6  -2.92  [1] |
| Kisii DGH | 5933.9  165.39  [35, 32]  (31) | 6188.9  171.91  [33, 31]  (29) | 4.3  6.52  [-2] |
| **Coastal** |  |  |  |
| Kilifi DGH | 3408.1  94.67  [17,14]  (5) | 4165.8  122.52  [20, 16]  (11) | 22.2  27.85  [6] |
| Malindi DGH | 2864.2  79.56  [16, 13]  (7) | 3436.8  95.47  [18, 16]  (20) | 20.0  15.91  [13] |
| Msambweni DGH | 1766.0  49.73  [9, 7]  (0) | 2615.7^4^  72.66  [13, 8]  (1) | 48.1  22.9  [1] |

**Note:**

^1^ A single meteorological station was used for the hospitals immediately surrounding Lake Victoria, based at Kisumu, this met station had complete records for the period of observation and there were no other met stations located close to the catchment of the Siaya hospital. This station is however located close, 41km, to the Siaya catchment area.

^2^ Relative changes to describe changes between period one and two and it compares the absolute change in total precipitation to the reference value which here is the value of the estimate in the first period

^3^ Absolute change to describe the actual increase or decrease in the number of continuous months with rainfall >60mm

^4^ Rainfall data for 2009 were missing for Bungoma and Msambweni and was obtained from the nearest possible metrological station with complete data

**AI Table S2:** Summary of *Pf*PR survey data showing the median age corrected parasite prevalence estimates (including the inter-quartile range), number of survey data points across different sites by the two time periods. Estimates exclude data from surveys where less than 50 people were examined; 24 surveys excluded in the first period and 102 surveys excluded in the second period.

| **Hospital Location** | **Median Age-Corrected Parasite Prevalence [IQR]**  **(Number of surveys included)**  **Jan 2002-Aug 2006** | **Median Age-Corrected Parasite Prevalence [IQR]**  **(Number of surveys included)**  **Sept 2006-Feb 2010^1^** | ***Relative Change (%)***  **[Absolute Percentile Change]^2^**  **(95% CI)** |
| --- | --- | --- | --- |
| **Western/Lakeside** |  |  |  |
| Bungoma DGH | 15.3%  [12.3-20.2]  (9) | 44.9%  [39.7-63.9]  (9) | 193.5  [35.9]  (22.0 - 51.6) |
| Kisumu DGH | 70.6%  [36.7-80.7]  (8) | 29.8%  [13.6-52.2]  (17) | -57.8  [-25.4]  (-57.9 - -3.5) |
| Siaya DGH | 54.7%  [46.8-56.9]  (6) | 46.5%  [37.2-60.0]  (13) | -15.1  [-6.8]  (-19.6 - 7.7) |
| **Highlands** |  |  |  |
| Kericho DGH | 5.2%  [NA]  (1) | 0.01%  [0.01-1.0]  (7) | -99.8  [-5.2]  (-5.2 - -0.3) |
| Kisii DGH | 22.2%  [16.3-26.0]  (4) | 1.9%  [0.01-5.7]  (49) | -91.4  [-19.1]  (-24.1- -10.8) |
| **Coastal** |  |  |  |
| Kilifi DGH | 12.9%  [4.8-48.9]  (19) | 3.7%  [0.01-11.5]  (45) | -71.1  [-8.9]  (-24.4 - -2.2) |
| Malindi DGH | 11.8%  [6.3-40.9]  (11) | 4.5%  [1.6-6.7]  (16) | -61.9  [-6.9]  (-35.6 - 0.04) |
| Msambweni DGH | 10.4%  [0.8-27.4]  (8) | 11.8%  [4.5-19.8]  (16) | 13.5  [1.2]  (-18.5 -11.9) |

**Notes:**

^1^Includes data from school surveys done in January and February 2010 in Bungoma, Kisumu, Siaya and Kericho areas

^2^95% confidence interval(s) for percentile difference(s) between values of pfpr210 in first and second groups

**AI Table S3:** Characteristics of hospital-catchment sites - Summary of the ITN coverage across different sites. The data was extracted from clusters located within the catchments of selected hospitals. ITN use defined as proportion of the population of all ages who reported using an ITN the night before the survey.

| **Hospital** | **ITN use**  **Sept-Dec 2005**  **(PSI TrAC)**  **Number using ITN [Number Examined]**  **Number Clusters**  **Sept 2003-Aug 2006** | **ITN use**  **July-Oct 2007**  **(MIS & PSI TrAC)**  **Number using ITN [Number Examined]**  **Number Clusters**  **Sept 2006-Aug 2009** | ***Relative Change (%)^1^***  **[Absolute Change ]**  **(95% CI)^2^** |
| --- | --- | --- | --- |
| **Western/Lakeside** |  |  |  |
| Bungoma DGH | 20.5%  74[361]  7 | 36.0%  542[1504]  13 | 75.6  [15.5]  (10.7-20.3) |
| Kisumu DGH | 26.2%  37[141]  6 | 47.3%  637[1346]  16 | 80.5  [21.1]  (13.4-28.8) |
| Siaya DGH | 21.0%  17[81]  4 | 38.8%  109[281]  3 | 84.8  [17.8]  (7.3-28.3) |
| **Highlands** |  |  |  |
| Kericho DGH | 14.3%  28[196]  6 | 32.0%  285[891]  10 | 123.8  [17.7]  (11.9-23.5) |
| Kisii DGH | 35.6%  57[160]  8 | 51.1%  519[1015]  9 | 43.5  [15.5]  (7.5-23.5) |
| **Coastal** |  |  |  |
| Kilifi DGH | 20.5%  31[151]  4 | 38.1%  340[893]  8 | 85.9  [17.6]  (10.4-24.8) |
| Malindi DGH | 8.8%  24[274]  5 | 49.9%  202[405]  4 | 467.0  [41.1]  (35.2-47.0) |
| Msambweni DGH | 15.1%  36[238]  5 | 38.4%  339[883]  7 | 154.3  [23.3]  17.7-28.9) |

**Notes:**

^1^ Relative change to describe changes between period one and two and it compares the absolute change in ITN coverage to the reference value which here is the value of the estimate in the first period

^2^ Absolute change to describe the actual increase or decrease in ITN coverage between period 1 and 2 and the 95% CI

**AI Table S4:** Characteristics of hospital-catchment sites - Summary of ITN per capita distribution * across different study sites using population estimates from with the catchment areas

| **Hospital** | **Projected Population**  **2006^1^** | **Per capita ITN distribution^2^**  **[Total ITNs]**  **Oct 2004- Aug 2006** | **Projected Population**  **2009^1^** | **Per capita ITN distribution^2^**  **[Total ITNs**  **Sept 2006- Aug 2009** |
| --- | --- | --- | --- | --- |
| **Western/Lakeside** |  |  |  |  |
| Bungoma DGH | 760,871 | 0.32  [246,852] | 861,521 | 0.44  [377,098] |
| Bondo DGH | 174,238 | 0.29  [50,239] | 183,782 | 0.51  [93,919] |
| Kisumu DGH | 635,677 | 0.44  [279,354] | 681,484 | 0.69  [468,589] |
| Siaya DGH | 259,105 | 0.52  [135,610] | 266,265 | 0.70  [186,615] |
| **Highlands** |  |  |  |  |
| Kericho DGH | 483,176 | 0.23  [108,725] | 520,365 | 0.50  [258,749] |
| Kisii DGH | 467,209 | 0.34  [156,678] | 496,544 | 0.53  [263,216] |
| **Coastal** |  |  |  |  |
| Kilifi DGH | 420,051 | 0.23  [95,318] | 460,990 | 0.53  [246,251] |
| Malindi DGH | 255,049 | 0.49  [124,688] | 282,426 | 0.80  [227,291] |
| Msambweni DGH | 289,883 | 0.14  [40,520] | 313,399 | 0.43  [133,198] |

**Notes**

* *ITN Process Data:* First annual sales figures from PSI for the retail sector, social marketing campaigns were available by region. Yearly data for each region has been divided by the number of districts in each region in which PSI carried out its activities to obtain district specific estimates for all the nine sites. These were then distributed equally in each month of each year and these were obtained for the period September 2003 through to October 2004. Secondly data on monthly sales of heavily subsidized ITN by geo-positioned clinics in each of the nine defined catchment areas were available for the period October 2004 to December 2009. Finally net distribution volumes from the large-scale free distribution campaign of free ITN to children under the age of five years were recorded per geo-located distribution point within each hospital catchment area. Cumulative monthly ITN distribution volumes per site were computed per capita using population size estimates specific to the catchment area defined for each facility.

^1^Populations projected from the 1999 national census to 2006 and 2009 using district-specific inter-censal natural growth rates

^2^Cumulative monthly ITN distribution volumes expressed per capita across the 9 hospital catchment areas in August 2006 and August 2009
